# Barriers to and facilitators of hypertension management in Asmara, Eritrea: patients’ perspectives

**DOI:** 10.1186/s41043-017-0090-4

**Published:** 2017-04-13

**Authors:** Merhawi Teklezgi Gebrezgi, Mary Jo Trepka, Eyob Azaria Kidane

**Affiliations:** 1 Department of Epidemiology, Robert Stempel College of Public Health and Social Work, Florida International University, 11200 SW 8th Street, Miami, FL USA; 2 Asmara College of Health Sciences, School of Public Health, PO Box 8566, Asmara, Eritrea

**Keywords:** Hypertension, Barriers, Management, Qualitative, Eritrea

## Abstract

**Background:**

Personal hypertension management is a cornerstone in the prevention of hypertension complications. In Eritrea, the increase in the national life expectancy rate has been accompanied by an increase in hypertension complications such as stroke. Hence, this study was designed to identify barriers and facilitates to hypertension management from the perspective of the patients.

**Methods:**

This was a qualitative study of a total of 48 individual in-depth interviews and two focus group discussions. It was conducted among hypertensive patients who were attending outpatient services at two hospitals in Asmara, Eritrea.

**Results:**

This study identified barriers and facilitators of hypertension management related to the individual patient, family and community, and healthcare system. With respect to individual factors, economic barriers, stress, non-adherence to medications due to the use of traditional remedies, and difficulties and misconceptions about following physical activity guidelines were mentioned as barriers to hypertension management. Related to the community and healthcare system, low community awareness, community stigma, and inadequate health promotion materials were stated as barriers. Individual knowledge, family, and government support were reported as very important factors to the patient’s success in the personal hypertension management.

**Conclusions:**

Counseling patients about adherence to medication, strengthening family and government support, and empowering families and the community with appropriate knowledge of hypertension management could potentially help in an individual’s adherence.

## Background

Globally, hypertension is one of the most widely prevalent diseases [1]. The estimated prevalence of hypertension among the adult world population was 26.4% (972 million) in 2000 with an estimated prevalence of 29.2% (1.5 billion) in 2025 [2]. Therefore, it is becoming a global public health threat, consuming a large percentage of public health expenditures [3]. Although its prevalence varies among countries, it is increasing in developing countries [4–9]. In the African continent, the overall prevalence of hypertension has been difficult to determine because a significant segment of its population is still afflicted by severe poverty, famine, and civil strife [5].

Patients with hypertension are at increased risk of cardiovascular- and cerebrovascular-related deaths. The majority of heart-related deaths are attributable to elevated blood pressure [10–12]. Therefore, hypertension management is critical to the prevention of cardiovascular and cerebrovascular events.

Personal management of hypertension is the cornerstone of hypertension care and includes practicing a healthy lifestyle and adherence to anti-hypertension medication. Physical activity, healthy diet, restricted alcohol consumption, salt restriction, and avoidance of tobacco use and stress are the recommended healthy lifestyle choices in hypertension care [13, 14]. Unhealthy lifestyle choices and non-adherence to medication have been challenges in the continuum of hypertension care resulting in poor health outcomes, low quality of life, and unwanted health care costs. There are different personal and environmental factors that facilitate and hinder healthy lifestyle choices for an individual with hypertension. The individual with hypertension, the family of the patient, the community, and the healthcare provider play important roles [15–17]. Thus, the patient and the family should have adequate knowledge of hypertension and its management. The care provider should also tailor messages in a way the patient understands.

Eritrea is located in the horn of Africa. The population of Eritrea has grown from 3.5 million in 2000 to 5.8 million in 2014, and life expectancy has increased from 54.4 in 2000 to 64.7 in 2014 [18]. The country is categorized as one of the least developed countries by the World Bank [19]. With the increase in life expectancy, chronic diseases and their complications are expected to rise. Stroke was the number one cause of death in Eritrea (139 per 100,000 population) accounting for 7.1% of all deaths in 2014 [18]. The existing healthcare system prioritizes maternal and child health to achieve the millennium development goals and has scored remarkable achievements, but little attention is given to noncommunicable diseases like hypertension. Previously published studies about the status of hypertension in the Eritrean population have reported about the prevalence and awareness of hypertension or its risk factors in the community [9, 20, 21]. One study has tried to see the pattern of antihypertensive drugs among patients with cardiac complications [22]. What remains unknown is the extent to which patients in Eritrea are able to adopt the necessary lifestyle changes and adhere to their medication and what factors facilitate or hinder effective personal hypertension management. Therefore, this study was designed to identify barriers and facilitates to the hypertension management from the perspective of the patients.

## Methods

This was a qualitative study of in-depth interviewers and focus groups conducted among hypertensive patients who were attending outpatient and follow-up services at the Halibet National Referral Hospital and Hazhaz Regional Referral Hospital in Asmara, Eritrea. These hospitals were chosen because they include hypertension follow-up centers as part of their outpatient service. In these hospitals (hypertension follow-up centers), patients attend a follow-up visit every 3 months. Health education is given on a daily basis at the outpatient service, and individual patients receive counseling at the doctor’s office. A total of 48 hypertensive patients were interviewed. Subjects were selected from the list of daily patients at the hypertension follow-up centers. A random starting point was selected, and a fixed periodic interval was used. The total number of daily visitors was divided by the required sample size for each day to get the interval. Following this procedure, an in-depth interview was conducted after an informed consent was obtained from the subjects.

The interview was based on a pre-tested interview checklist which included closed- and open-ended questions. The closed-ended questions were related to demographic information (age, sex, ethnicity, address, religion, and education), and the open-ended questions were related to awareness and practice of the patients regarding hypertension management. Participants were asked about their experiences living with hypertension, knowledge, and perception of the principles of hypertension management; the practice of lifestyle modifications; barriers and facilitators to hypertension management; sources of information; their interaction with family, community, and healthcare providers; and their opinion on how the prevention activities should be structured.

In addition to the interviews, two separate male and female focus group discussions (FGDs), each consisting eight participants (aged 30–60 years), were conducted. The FGDs were moderated by a facilitator, and the discussions were open until a saturation point was reached for every topic raised. The FGDs lasted for 1 to 2 h. Findings of the discussions were noted by two separate reporters and classified into the following main themes: individual barriers and facilitators to hypertension management (e.g., knowledge, practice, and economic status), community (e.g., family and community awareness), and healthcare system-related factors.

## Results

Fifty-four percent of the study participants were female. The mean age of the in-depth interview and focus group participants was 61 ± 7 and 53 ± 4 years respectively. Table 1 provides the demographic information of the study participants.

**Table 1:** Demographic characteristic of in-depth interview participants

Participants’ characteristics	Response (*N* = 48) *n* (%)
Sex	
Male	26 (54.2)
Female	22 (45.8)
Age	
25–39 years	3 (6.2)
40–59 years	20 (41.7)
≥60 years	25 (52.1)
Education	
Illiterate	4 (8.3)
1–5 grade	27 (56.3)
≥6 grade	17 (35.4)
Religion	
Christian	28 (58.3)
Muslim	20 (42.7)
Ethnicity	
Tigrinya	31 (64.6)
Tigre	8 (16.7)
Saho	3 (6.2)
Other	6 (12.5)
Residence	
Urban	32 (66.7)
Rural	16 (33.3)
Duration of stay with hypertension	
1–2 years	16 (33.3)
3–6 years	27 (56.3)
≥7 years	5 (10.4)

We identified a total of nine barriers and six facilitators. Barriers and facilitators were categorized into three themes, namely, individual, community, and health system level factors (Table 2).

**Table 2:** Frequency of barriers and facilitators of hypertension management

Theme categories			*n* (%)
Individual level	Barrier	Misconception about physical activity (difficult to implement, can be harmful to the body)	22 (45.8)
		Stress (family burden, lifelong nature of treatment, lifestyle-related, fear of complication)	32 (66.7)
		Use of traditional medicine	29 (60.4)
		Financial constraints	33 (68.8)
	Facilitator	Knowledge about hypertension management (mentioned three or more factors)	42 (87.5)
Community level	Barrier	Community stigma	14 (29.1)
		Low community awareness	26 (54.1)
	Facilitator	Family involvement	
		Social interaction	
Health system level	Barrier	Related to health professional (new doctors, high workload)	28 (58.3)
		Inadequate health promotion (low-effect posters and low media coverage)	18 (37.5)
		Few number of hypertension follow-up centers	12 (25.0)
	Facilitator	Devoted health professionals	35 (72.9)
		Good communication with their doctors	30 (62.5)
		Government support (medications)	40 (83.3)

### Individual barriers and facilitators

An individual facilitator of effective hypertension management was that participants had a good understanding of what they needed to do. They mentioned adherence to medication, salt consumption reduction, physical activity, diet control, and lowering of alcohol consumption as key components of hypertension management. They noted that practicing these activities requires “self-discipline.”

There were misconceptions and difficulties in implementing physical activity recommendations. Physical activity was reported to be the most difficult recommendation to be implemented. The most common exercise they practiced was “walking,” and walking was mentioned as the easiest way to achieve the desired prevention. Some of the participants did not acknowledge the importance of physical activity. They stated that they were too old for physical activity and doing so could bring more damage to their body.

Sometimes physical activity can hurt if you do it at older age. Because our bones are not resistant to external power. (Response from focus group discussion)

Stress was also reported as a barrier. Participants stated that hypertensive patients are prone to stress. Hypertensive patients have the responsibility of caring their families. They reported that the lifelong nature of the treatment, rules about diet and drinking, fear of complications, and lifestyle modifications create additional stress to them. The participants also related that “stress increases workload to the heart” which they saw as a possible risk factor for hypertension complications.

I take care of the whole family…this creates stress on me. (Response from in-depth interview)

Another barrier was the use of traditional medicine. Sixty percent of the participants had practiced traditional medicine in the past. Herbal medicine was the most commonly used remedy. Participants obtain information about those practices from their friends. Participants also reported that they quit pharmacological medication when they were practicing traditional medicine. The “lifelong nature,” “side-effects,” and “ineffectiveness” of antihypertensive medications were mentioned to be the most important reasons for using traditional medicine in place of prescribed antihypertensive medication. Participants also reported that there were advertisements about the effectiveness of some traditional remedies. For example, some shops had advertisements about *Moringa* plant products as a cure for hypertension.

Participants reported financial constraints inhibiting their ability to follow the recommended diet management. They stated that the cost of fruits is too high in the market, and the diet recommendations did not consider their economic status. However, some participants argued that even though there were some economic problems, there were enough cheap vegetables and fruits in the market which were adequate to manage hypertension. Some participants described poverty to have a positive effect on the hypertension management. According to their perception, poor patients are not at risk of becoming obese because of their economic status. In contrast to this, others argued that money is needed to manage hypertension adequately.

I don’t have blood pressure cuff at home. I am poor and so I can’t afford it. (Comment from focus group discussion)

### Community-level barriers and facilitators

Participants highly valued the participation of the community in the hypertension management and discussed ways of improving the awareness of their families in particular and the community in general. However, they stated that the knowledge of the community regarding hypertension management is low, and they recommended efforts be made to increase community awareness about hypertension and its complications.

Participants in this study acknowledged the involvement of their families in the hypertension management. Families are the key stakeholders in hypertension management according to the participants. They described families as “home doctors” and highlighted the importance of having adequate knowledge among the members of the family. Participants mentioned that families can either be directly involved in the hypertension management by deciding the food choices of the patients or indirectly by reminding the patient what to do.

Food choices of patients were determined by their family members. Therefore, adherence to diet recommendations was reported to be the easiest recommendation to be implemented. Some participants also reported disagreement with their family members because of the wrong diet choices.

My family doesn’t have enough knowledge about the diet management, therefore, I always argue with them. (Response from in-depth interview)

Some participants reported that they come to the hypertension follow-up center with one of their family members because they need somebody to care them. In this way, some family members participate in the health education program provided at the follow-up center. The participants called for the strengthening of such activities.

My daughter brought me here, and when she heard what the doctor was saying in the health education session, she was really surprised. (Comment from focus group discussion)

Participants claimed that social interaction with friends and other members of the community is one of the means to get information about hypertension and its management. The unavailability of a common meeting center with their friends was mentioned as a barrier. In this regard, some participants suggested having a “social center” for patients with hypertension. According to them, this would be beneficial for the practice of behavioral lifestyle modifications and sharing of ideas and opinions about hypertension and its complications.

I find most of my friends in Bar Vitoria (place of gathering mainly for the elderly people) and I used to discuss hypertension and its complications in that place. (Comment from focus group discussion)

Participants also reported stigma that they experienced as patients with hypertension. Participants explained that the perception of some community members to consider hypertensive patients as non-productive puts pressure on them. They also reported that community considers hypertension as a disease of the “old and wealthy people.”

Some people consider me as disabled and they don’t give me any task to do and this adds stress. (Response from in-depth interview)

### Healthcare system-related barriers and facilitators

Health professionals were mentioned to be the key source of information for hypertensive patients. Patients reported having good communication with their doctors. However, they stated that with new doctors, there are delays in establishing comfortable communication between the patient and care provider. Moreover, the high workload of doctors hinders detailed discussion between the patient and the doctors. Although participants had some complaints about some doctors, considering the workloads they have, doctors were generally described to be cooperative.

I always go to my doctor with lots of questions and always he is happy to answer all. (Response from in-depth interview)

Participants appreciated the government’s support in terms of medication and reported that they get free medication for the hypertension management. They reported that they follow their medication in a 3-month appointment interval, and if they feel something unusual, they are free to see their doctor at any time during the morning hours of the working days.

I am grateful to the government for making hypertension medication free of charge. If it wasn’t like this, couldn’t we have died at the time we were diagnosed? (Comment from focus group discussion)

On the other hand, participants did not think that there were enough health promotion activities conducted by the Ministry of Health about hypertension and its management. They reported that the television and the radio programs have little coverage about the disease. Participants also expressed their concern about Ministry of Health posters. They reported that there are few posters about hypertension, and most of them are difficult to understand. They stated that the posters did not look as if they were prepared with much input from target audiences. They suggested that the Ministry of Health produce more posters and consider the target audience. Moreover, participants recommended opening more hypertension follow-up centers in places where there are no hypertension follow-up centers.

## Discussion

To the best of our knowledge, this study is the first study on barriers to and facilitators of the personal hypertension management in Eritrea. We were able to identify barriers to and facilitators of hypertension management related to the individual, family and community, and healthcare system. Related to individual factors, the key findings were that participants reported stress related to their diagnosis and difficulties and misperceptions about following physical activity guidelines. Furthermore, there were economic issues that inhibited their ability to follow diet-based recommendations and obtain blood pressure cuffs to monitor their blood pressure. In addition, participants reported the use of traditional remedies which may interfere with their antihypertensive medication adherence or possibly interact with their medication. Related to family and community factors, family member support was seen as very important to the patient’s success in personal management, and low level of awareness about hypertension in the community and community stigma affect the patients. With respect to the healthcare system, there were few concerns about their medical care, but the participants thought that the Ministry of Health should provide more education about hypertension and increase awareness of the community. The main reported facilitators of hypertension management were individual knowledge about hypertension, family support, the dedication of healthcare professionals, and free supply of antihypertensive medication.

Control of blood pressure is the ultimate goal of all hypertension management activities. Patient’s knowledge and practices are important so as to achieve the desired blood pressure level to control complications of hypertension. Although a quantitative study is required, participants in this study appeared to have adequate knowledge about hypertension and its management. Health education sessions are given to patients on a continuous basis at the hypertension follow-up centers. In addition, hypertensive patients receive individual counseling at the doctor’s office as part of their regular follow-up. This could be a reason for the relatively high level of knowledge of the patients. Therefore, the counseling and health education activities at the hypertension follow-up centers should continue. Moreover, expanding health education sessions to other hospitals and hospital wards could be beneficial. Some of the misconceptions about physical activity could be due to the misinterpretation of the health education message. Patients may perceive “physical activity” as rigorous exercise. Therefore, the health education given at those facilities should be given in clear and understandable language to avoid misconception among patients.

Adherence to medication is the key to hypertension management. In this study, participants reported using traditional medicine with subsequent cessation of antihypertensive medication. This study and other studies in Nigeria and India have reported about the practice of alternative medicine among the hypertensive patients [23, 24]. Non-adherence to antihypertensive medication results into hypertensive complications. Studies have shown that there is a steady rise in disease burden of heart failure, myocardial infarction, and stroke in Eritrea [9, 20].Therefore, healthcare providers need to ask patients about the use of traditional medicine, determine if there may be interactions between the traditional medicine and the antihypertensive medication, and discuss the importance of adherence to antihypertensive medication. Moreover, the Eritrean government needs to regulate the content of advertisements of remedies to prevent the dissemination of inaccurate or misleading information.

Family members in particular and the community in general are stakeholders in the hypertension management system. Participants in this study mentioned a family to be the foundation of hypertension management. Since family members are involved in influencing the lifestyle of the patients, health education program about hypertension should involve the general community in general and family members of the hypertensive patients in particular. Moreover, the Ministry of Health could adopt the peer education approach to increase the knowledge and practice of personal hypertension management. Studies in developing countries have shown the effectiveness of peer education in noncommunicable disease management [25, 26].

The economic status of the patient can affect hypertension management in different ways. In some areas, non-adherence to medication is one of the main effects of low economic status due to difficulties covering the costs of drugs [27, 28]. As opposed to the findings of those studies, antihypertensive drugs are provided free of charge in Eritrea. Therefore, in Eritrea, economic status does not seem to influence the adherence of patients to antihypertensive medications. However, the economic status does influence patients’ adherence to lifestyle modifications such as diet management and buying blood pressure management apparatus as reported by the participants. Health education needs to address how to adhere to a healthy diet using food items that are economical.

In addition to the individual- and community-level barriers, participants also identified barriers to and facilitators of hypertension management related to the health services and policy. Although participants identified some barriers, the majority of the points mentioned were facilitators of management. A study conducted in low-resource settings indicated that Eritrea was one of the countries identified with deficits in health financing, access to basic technologies and medicines, medical information systems, and the health workforce to combat noncommunicable diseases. In spite of this, the study indicated that Eritrea has dedicated staff in primary healthcare facilities [29]. The later strength was also reported in this study. The fact that drugs are given free of charge, given Eritrea is a country described with a deficit in health financing, could show the government’s commitment towards combating hypertension. This study also highlighted a positive patient and provider interaction as an important asset in hypertension management. Therefore, the health system can be considered as an asset in the hypertension management.

The media and health promotion materials are important dimensions to advocate for disease prevention and management. From the findings of this study, it can be recommended that the Ministry of Health should give due attention to coverage of hypertension topics in the media. Moreover, the ministry should increase the production of, and involve communities in the design of, posters, leaflets, and other written health promotion materials. These activities could have a significant impact on increasing knowledge in the general community and to introduce healthy behavioral lifestyle modifications among the hypertensive patients [30]. Distance to healthcare facility also affects the adherence of patients to medications [15]. Therefore, providing hypertension centers in remote locations could potentially improve hypertension management.

The qualitative nature of the study was helpful in identifying barriers that might not be ascertained in surveys, in understanding patient perceptions, and in elucidating patients’ perspectives about hypertension management principles. Despite this advantage, there were certain limitations worth mentioning here. First of all, the qualitative nature of the study prevents conclusions about how common some of the barriers are and does not allow analysis of the association between particular factors and demographic variables. Second, the reported nature of some practices was also subjected to recall bias. Third, since this study was conducted in two hospitals in the capital city, Asmara, it may not be possible to generalize to other hospitals in the country. Nevertheless, this study adds to the limited data available and provides important findings regarding the obstacles to successful hypertension management.

## Conclusions

This study has identified barriers to and facilitators of hypertension management among the hypertensive population at the individual, family and community, and healthcare system levels. Based on the results of this study, counseling patients about care in the use of traditional medicine, appropriate physical activity, and how to adhere to diet recommendations with affordable food choices could potentially increase their adherence to the principles of hypertension management. Moreover, empowering families and community with adequate knowledge about hypertension and its management and continued government support with medications could help adherence. Finally, it is important to further explore the findings of this study with a quantitative study to ascertain how common some of the factors mentioned by the participants are.
